# RsmA Regulates Biofilm Formation in *Xanthomonas campestris* through a Regulatory Network Involving Cyclic di-GMP and the Clp Transcription Factor

**DOI:** 10.1371/journal.pone.0052646

**Published:** 2012-12-21

**Authors:** Xiu-Hong Lu, Shi-Qi An, Dong-Jie Tang, Yvonne McCarthy, Ji-Liang Tang, John Maxwell Dow, Robert P. Ryan

**Affiliations:** 1 State Key Laboratory for Conservation and Utilization of Subtropical Agro-bioresources, and College of Life Science and Technology, Guangxi University, Nanning, People’s Republic of China; 2 Department of Microbiology, BioSciences Institute, University College Cork, Cork, Ireland; University Medical Center Utrecht, The Netherlands

## Abstract

Biofilm formation and dispersal in the black rot pathogen *Xanthomonas campestris* pathovar *campestris* (*Xcc*) is influenced by a number of factors. The extracellular mannanase ManA has been implicated in biofilm dispersal whereas biofilm formation requires a putative glycosyl transferase encoded by the *xag* gene cluster. Previously we demonstrated that the post-transcriptional regulator RsmA exerts a negative regulatory influence on biofilm formation in *Xcc*. Here we address the mechanisms by which RsmA exerts this action. We show that RsmA binds to the transcripts of three genes encoding GGDEF domain diguanylate cyclases to influence their expression. Accordingly, mutation of *rsmA* leads to an increase in cellular levels of cyclic di-GMP. This effect is associated with a down-regulation of transcription of *manA*, but an upregulation of *xag* gene transcription. Mutation of *clp*, which encodes a cyclic di-GMP-responsive transcriptional regulator of the CRP-FNR family, has similar divergent effects on the expression of *manA* and *xag*. Nevertheless Clp binding to *manA* and *xag* promoters is inhibited by cyclic di-GMP. The data support the contention that, in common with other CRP-FNR family members, Clp can act as both an activator and repressor of transcription of different genes to influence biofilm formation as a response to cyclic di-GMP.

## Introduction

The formation, maturation and dispersal of biofilms are key processes within the lifestyle of many bacteria including animal and plant pathogens [1], [2]. Biofilm formation is important for many pathogenic organisms, as bacteria within these multicellular structures are often considerably more resistant to diverse stresses that can include the action of host defences [1], [2]. Equally the release of bacteria from biofilms is significant for the progression of disease into uninfected tissue and for completion of the disease cycle [3]. The extracellular environment undoubtedly influences many aspects of bacterial behaviour including the dynamics of biofilm formation and dispersal. An array of signal transduction systems link the sensing of specific environmental cues to appropriate alterations in bacterial physiology and/or gene expression. An understanding of these different regulatory elements or pathways, how they are integrated and how they act to influence biofilm dynamics may have important implications for the control of bacterial disease.

*Xanthomonas campestris* pathovar *campestris* (hereafter *Xcc*) is the causative agent of black rot disease of cruciferous plants, which is an important disease globally [4]. A number of factors and several regulatory pathways have been implicated in the formation or dispersal of biofilms by *Xcc* [5]. Biofilm formation requires the synthesis of the extracellular polysaccharide xanthan and an uncharacterised polysaccharide whose synthesis is directed by the products of the *xag* gene cluster [6]. Conversely, the extracellular enzyme beta (1,4)-mannanase has been implicated in biofilm dispersal [5], [6].

Cell-to-cell signalling mediated by the diffusible signal molecule DSF (for Diffusible Signal Factor; *cis*-11-methyl-dodecenoic acid) regulates biofilm dispersal [5]. Both the synthesis and perception of the DSF signal require products of the *rpf* gene cluster (for regulation of pathogenicity factors). The synthesis of DSF is dependent on RpfF, whereas the two-component system comprising the sensor kinase RpfC and regulator RpfG is implicated in DSF perception and signal transduction [7], [8]. RpfG is a regulator with a CheY-like receiver (REC) domain attached to an HD-GYP domain, which acts to degrade the second messenger bis (3′, 5′)-cyclic diguanosine monophosphate (cyclic di-GMP) [9], [10]. Mutation of *rpfF*, *rpfG*, or *rpfC* leads to an elevated level of cyclic di-GMP and the mutants exhibit an aggregative behaviour when grown in certain media, unlike the wild-type, which grows in a dispersed fashion [4], [5], [9], [11]. This is consistent with a body of work in a number of bacteria in which biofilm formation has been associated with elevated levels of cyclic di-GMP (reviewed recently by [12]–[16]).

The Rpf/DSF system positively influences the expression of the *manA* gene, encoding endo-mannanase, but negatively influences the expression of the *xag* gene cluster [6]. The regulatory effect of the DSF/*rpf* system on the expression of *manA* is believed to involve the transcriptional regulator Clp [17]. Elevated levels of cyclic di-GMP negatively influence the ability of Clp to bind to DNA [18], [19].

In previous work we have shown that the post-transcriptional regulator RsmA also influences biofilm formation in *Xcc.* RsmA is an RNA-binding protein functioning as a global regulator of various cellular processes in bacteria [20]. Deletion of *rsmA* in *Xcc* results in complete loss of virulence, a significant reduction in the production of the extracellular polysaccharide xanthan and extracellular enzymes including mannanase but an enhanced bacterial aggregation and cell adhesion. Mutation of *rsmA* does not alter the expression of *rpf* genes or the level of DSF in *Xcc* however, suggesting that the regulatory action of RsmA on biofilm formation is independent of cell-to-cell signalling under the conditions tested [21].

In this paper we address the mechanisms by which RsmA exerts a regulatory effect on biofilm formation in *Xcc*. In *Escherichia coli* and *Salmonella,* the RsmA homolog CsrA controls cyclic di-GMP metabolism [20], [22], [23]. Our initial finding that mutation of *rsmA* in *Xcc* also leads to an increase in levels of cyclic di-GMP prompted us to study the influence of RsmA on expression of genes in *Xcc* encoding GGDEF, EAL or HD-GYP domain proteins that are involved in cyclic di-GMP synthesis or degradation [12]. This allowed us to define a subset of these proteins whose expression is regulated by RsmA. Further to this we demonstrate by mutational analysis that Clp has opposite effects on the expression of *manA* and *xagA* genes. The work defines a regulatory network controlling the formation of biofilms in *Xcc* that comprises elements that are likely to be responsive to diverse environmental cues.

## Results

### Mutation of *rsmA* Leads to an Alteration in Biofilm Formation and Increase in Cellular Level of Cyclic di-GMP

**Figure 1:**
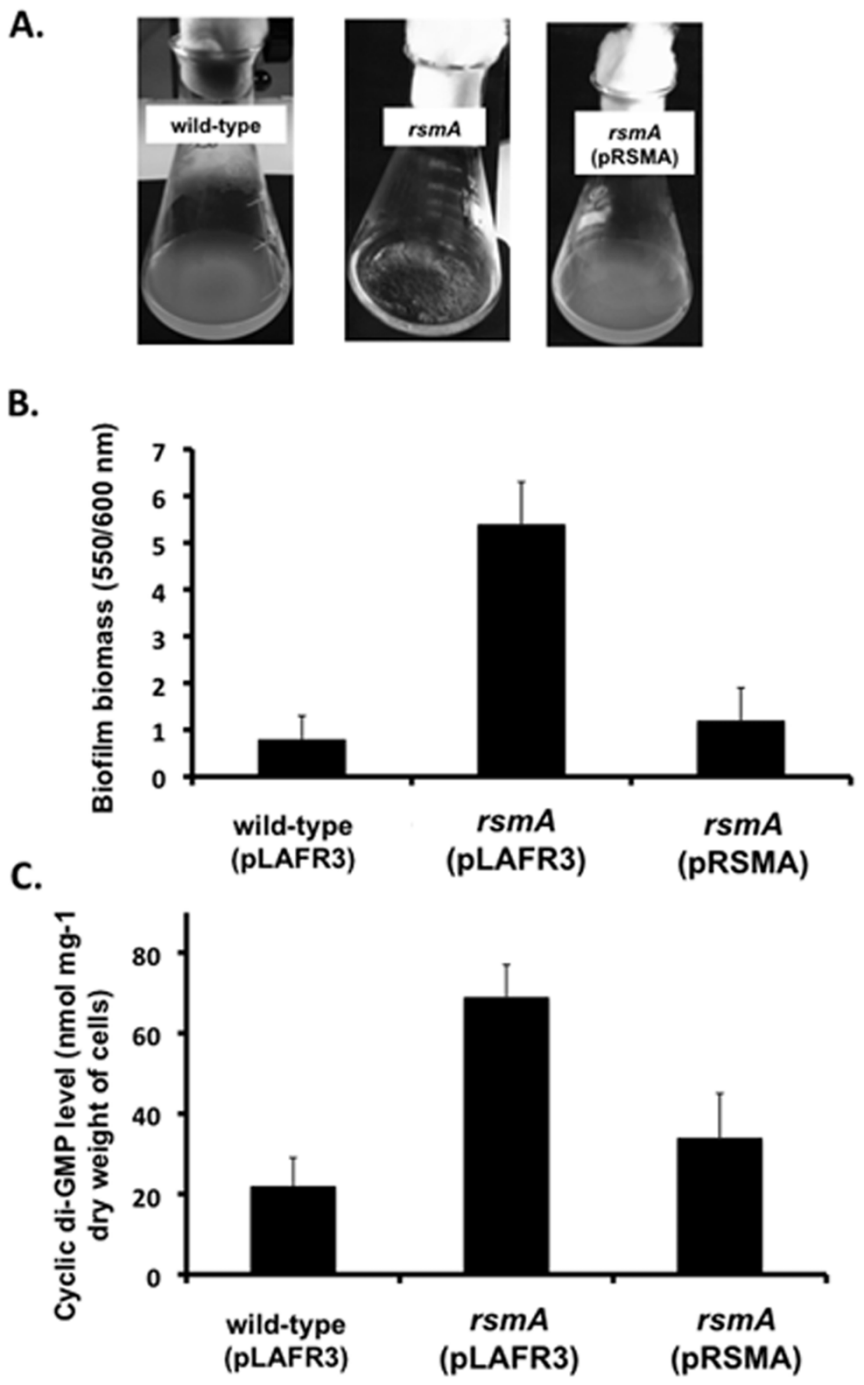
The *rsmA* mutant has an increased biofilm phenotype and is associated with an elevated intracellular level of cyclic di-GMP. (A) The *rsmA* mutant grows in an aggregated fashion in L medium, whereas the wild-type strain (8004) grows in a dispersed fashion. In trans expression of the gene encoding *rsmA* in the mutant restores the phenotype to wild-type. (B) The *rsmA* mutant also has increased biofilm and cell adhesion on a glass surface when assessed by crystal violet staining (see *Methods* for details). Values given are the mean and standard deviation of triplicate measurements. (C) Effects of mutation of *rsmA* on cyclic di-GMP level in *Xanthomonas.* Elevated levels of extractable nucleotide were seen after mutation of *rsmA*. Introduction of the cloned *rsmA* gene (pRSMA) into the *rsmA* mutant reduced cyclic di-GMP levels to wild-type. Values given are the mean and standard deviation of triplicate measurements (three biological and three technical replicates).

We have previously shown that mutation of *rsmA* in *Xcc* leads to an aggregative behaviour in liquid media and enhanced adhesion to glass surfaces [21]. This biofilm phenotype was assayed quantitatively using crystal violet staining to measure adherence to glass (see *Methods*). As expected, the *rsmA* mutant showed aggregative behaviour in liquid medium (Fig. 1A) and had higher levels of biofilm formation on glass than the wild-type (Fig. 1B). Complementation with *rsmA* expressed *in trans* restored the phenotypes to wild-type (Fig. 1A,B). In parallel, we measured the level of cyclic di-GMP in wild-type, *rsmA* mutant and complemented strains (see *Methods*). The cellular level of cyclic di-GMP in the *rsmA* mutant was elevated over that seen in the wild-type. Introduction of the cloned *rsmA* gene (pRSMA) into the *rsmA* mutant reduced the cyclic di-GMP level to wild-type (Fig. 1C).

### RsmA Binds to the Transcripts of Genes Encoding GGDEF Domain Proteins to Influence Expression

The finding that mutation of *rsmA* led to an increase in cellular levels of cyclic di-GMP prompted us to study the influence of RsmA on expression of all 37 genes in *Xcc* encoding proteins involved in cyclic di-GMP turnover: diguanylate cyclases with a GGDEF domain and phosphodiesterases with an EAL or HD-GYP domain [24]. The regulatory influence of RmsA on cyclic di-GMP metabolism could be exerted directly, through binding to transcripts of genes encoding these proteins, although more indirect regulation is certainly possible. In order to address these issues, we examined the effect of *rsmA* mutation on the transcription of genes encoding proteins with GGDEF, EAL or HD-GYP domains by qRT-PCR and also assessed the physical binding of RsmA to transcripts by electrophoretic mobility shift assays (EMSA).

**Figure 2:**
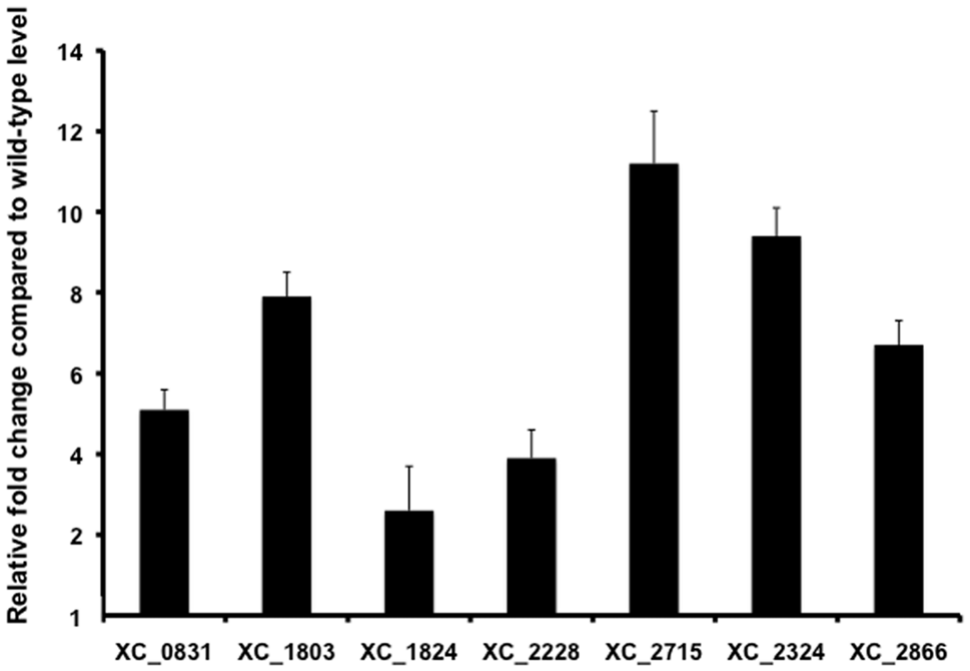
RsmA regulates the expression of genes encoding GGDEF and GGDEF/EAL domain proteins in *Xanthomonas.* Transcript levels of all genes encoding GGDEF, EAL or HD-GYP were measured in wild-type strain (8004) and *rsmA* mutant backgrounds by qRT-PCR. Bacterial strains were grown to an OD at 600 nm of 0.8 in complex medium before RNA extraction. All qRT-PCR results were normalized using the *Ct*s obtained for the 16S rRNA amplifications run in the same plate. The relative amounts of gene transcripts were determined from standard curves. Only the results from those genes (*XC_0831, XC_1803, XC_1824, XC_2228, XC_2324, XC_2715* and *XC_2866*) showing significant differences between the wild type and *rsmA* mutant are shown. Values given are the mean and standard deviation of triplicate measurements (three biological and three technical replicates). Similar differences were also seen when the bacteria were harvested at an OD at 600 nm of 1.0 and 1.5 (data not shown).

**Figure 3:**
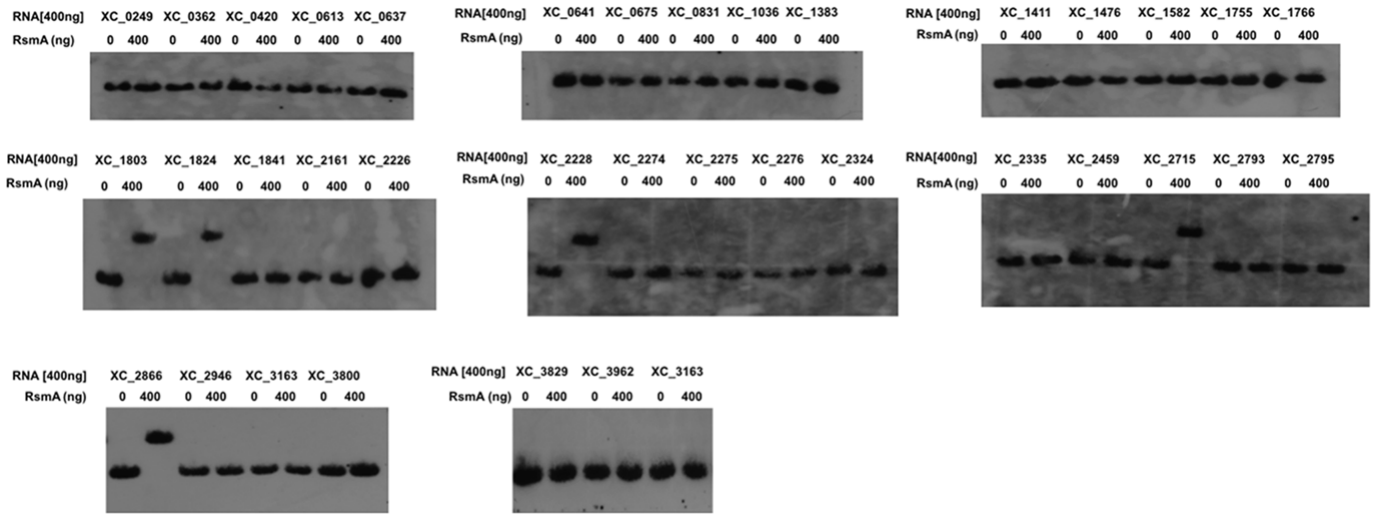
RsmA directly interacts with the transcripts of *XC_1803, XC_1824, XC_2228, XC_2715* and *XC_2866.* Electromobility shift assays were used to assess interaction between purified RsmA and *in vitro* synthesized transcripts of approximately 500 bp comprising the upstream region and a small part of the coding region of all genes that encode GGDEF, EAL or HD-GYP proteins (see *Methods* for specific details). These analyses revealed interactions of RsmA with transcripts of *XC_1803, XC_1824, XC_2228, XC_2715 and XC_2866*. The concentration of RNA used (400 ng) is indicated at the top of the appropriate lanes.

Mutation of *rsmA* led to an alteration in the transcript level of seven genes (*XC_0831*, *XC_1803, XC_1824, XC_2228, XC_2715, XC_2324* and *XC_2866*), which were all elevated in the mutant compared to the wild-type (Fig. 2). These genes encode proteins with a GGDEF domain or with both GGDEF and EAL domains (Fig. S1). The EMSA assays showed that RsmA interacted directly with the transcripts of five of these genes (*XC_1803, XC_1824, XC_2228, XC_2715* and *XC_2866*). However RsmA did not interact with transcripts of *XC_0831* or *XC_2324* or with any gene whose transcript level was unaltered (Fig. 3). The mobility shifts were reversed by competition with unlabelled RNA of the same sequence, but not by unrelated RNAs (data not shown), which indicated that the interactions were specific.

**Figure 4:**
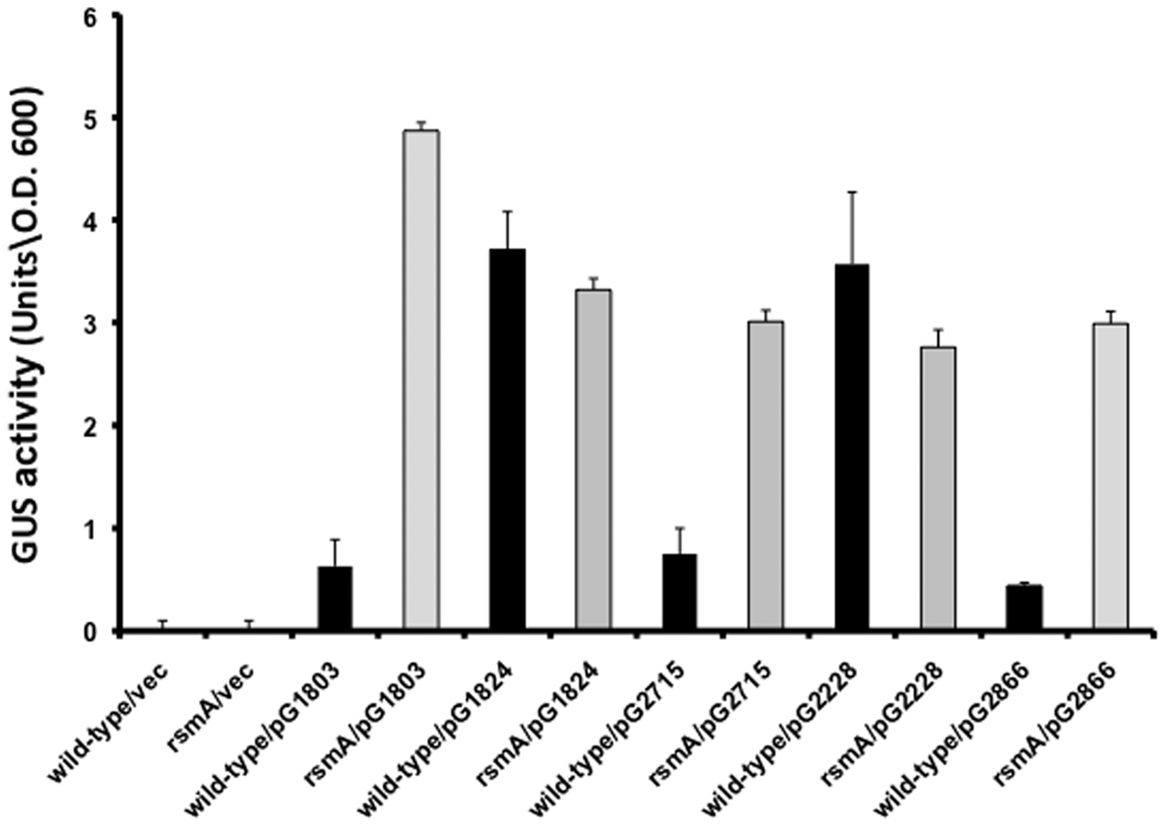
RsmA regulates the expression of the XC_1803, XC_2715 and XC_2866 proteins. Cells of the wild-type and *rsmA* mutant carrying different reporter plasmids were grown in complex media to an OD at 600nm of 1.0 and assayed for β-glucuronidase (GUS) activity as described in the *Methods*. Values given are means ± standard deviations of at least five independent experiments. Vec indicates the empty vector (control) plasmid. Similar effects were seen when the bacteria were harvested at an OD at 600 nm of 0.6 or 1.5 (data not shown).

The possibility that RsmA regulates the expression of *XC_1803, XC_1824, XC_2228, XC_2715* and *XC_2866* was further investigated using fusions to *gusA* (see *Methods*). Differences in the level of GusA expression between the *rsmA* mutant and wild-type were seen with fusions to *XC_1803*, *XC_2715* and *XC_2866*, which encode GGDEF domain proteins but not with fusions to *XC_1824* and *XC_2228,* which encode GGDEF/EAL domain proteins (Fig. 4). Taken together, the findings indicate that RsmA regulates expression of three GGDEF domain proteins and are consistent with the contention that regulation occurs at the post-transcriptional level. We speculate that RsmA binding promotes enhanced degradation of transcripts, thus reducing their relative level in the wild-type compared to the *rsmA* mutant. The mechanism by which RsmA affects the transcript levels of *XC_0831* and *XC_2324* is unclear however.

### Mutation of Genes Encoding Target GGDEF Domain Proteins Reduces the Level of Cyclic di-GMP in the *rsmA* Background

**Figure 5:**
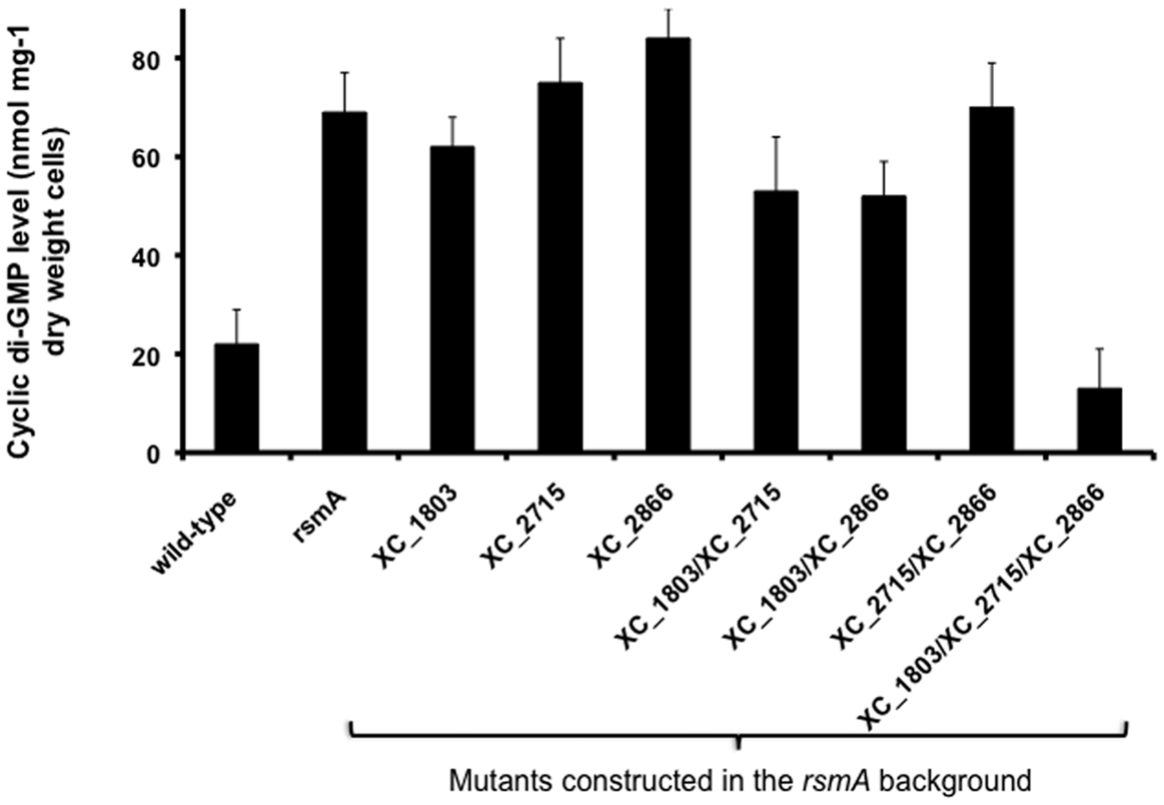
Combinatorial mutation of three genes encoding GGDEF domain proteins restores cyclic di-GMP levels of the *rsmA* mutant towards wild-type. The relative levels of cyclic di-GMP in the *rsmA* mutant background upon disruption of the *XC*_*1803*, *XC*_*2715* and *XC*_*2866* genes singly or in combination were determined as described in *Methods*. Only mutation of all three genes had a significant effect on cyclic di-GMP levels of the *rsmA* mutant. Values given are the mean and standard deviation of triplicate measurements (three biological and three technical replicates).

The elevated expression of the GGDEF domain proteins XC_1803, XC_2715 and XC_2866 in the *rsmA* mutant suggested that these putative diguanylate cyclases were responsible for the higher cellular level of cyclic di-GMP in this strain. In the first step towards testing this hypothesis, all three GGDEF domain proteins were purified as His6-tagged proteins and their activity in cyclic di-GMP synthesis was assessed (see Supporting Information). All three proteins were active as diguanylate cyclases (Fig. S2). Following this, the effect of mutation of *XC_1803, XC_2715* and *XC_2866,* both singly and in combination on the level of cyclic di-GMP in the *rsmA* mutant of *Xcc* was examined. The findings (Fig. 5) showed that although single mutations had no effect, double mutants reduced the level towards wild-type, while the triple mutation reduced the level of cyclic di-GMP in the *rsmA* mutant to wild-type levels. The triple mutation in the wild-type background had little effect on the cyclic di-GMP levels however. These findings link alteration in level of the three GGDEF domain proteins in the *rsmA* mutant to alteration in cyclic di-GMP.

### Mutation of Genes Encoding the GGDEF Domain Proteins XC_1803, XC_2715 and XC_2866 Alters Biofilm Formation by the *rsmA* Mutant

**Figure 6:**
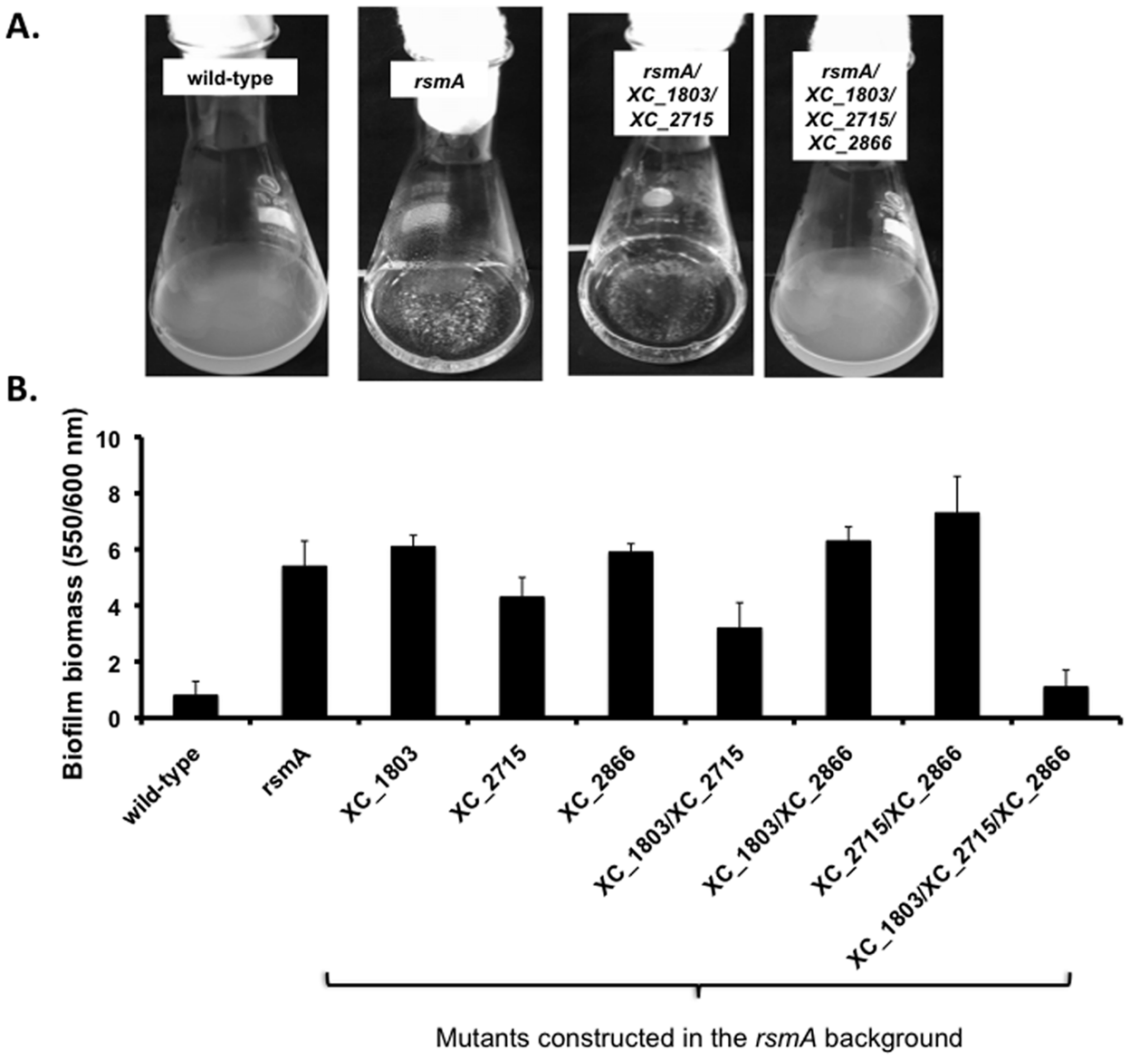
Combinatorial mutation of three genes encoding GGDEF domain proteins in the *rsmA* mutant background prevents aggregation and reduces biofilm formation towards wild-type. (A) A triple disruption of *XC*_*1803*, *XC*_*2715* and *XC*_*2866* background prevents aggregation of the *rsmA* mutant, although single or double mutants (such as *XC*_*1803*/*XC*_*2715* illustrated) have little or no effect. (B) A triple disruption of *XC*_*1803*/*XC*_*2715*/*XC*_*2866* in an *rsmA* mutant background leads to reduction in biofilm to a level similar to that of the wild-type strain as measured by the crystal violet staining assay (see *Methods* for details). In contrast mutation of *XC*_*1803*, *XC*_*2715* and *XC*_*2866* alone or in pairwise combination has little or no effect on biofilm formation in the *rsmA* mutant. Values presented are the means and standard deviations from triplicate measurements (three biological and three technical replicates).

As described above, mutation of *rsmA* leads to aggregation in liquid medium and an increased biofilm formation in glass tubes without shaking (Fig.1; [21]). To ascertain if the GGDEF domain proteins XC_1803, XC_2715 and XC_2866 contribute to these phenotypes of the *rsmA* mutant, double and triple mutants that were constructed in the *rsmA* mutant background (see above) were assessed for biofilm formation and aggregation. The triple mutation in the *rsmA* background gave rise to planktonic dispersed growth in liquid medium (Fig. 6A) and a similar biofilm phenotype to that of the wild-type (Fig. 6B). Double mutations showed no discernable difference in biofilm phenotype when compared to the *rsmA* mutant however (Fig. 6A, B). These findings indicate that the action of *rsmA* in the regulation of aggregation and biofilm formation is exerted through its influence on the expression of *XC_1803*, *XC_2715* and *XC_2866*.

### Cyclic di-GMP Influences Expression of *manA* and *xagA* Genes in a Clp-dependent Manner

The findings outlined above raise the question of how the elevation of levels of cyclic di-GMP in the *rsmA* mutant is linked to the formation of biofilms in *Xcc*. One possible link is through the cyclic di-GMP-responsive transcriptional regulator Clp (XC_0486). Previous studies have shown that Clp binds target DNA in the absence of any effector and that DNA binding is inhibited by cyclic di-GMP [25], [26]. Expression of the *manA* gene encoding the biofilm dispersing enzyme endo-mannanase is dependent on Clp [17] and is decreased in the *rsmA* mutant [21].

**Figure 7:**
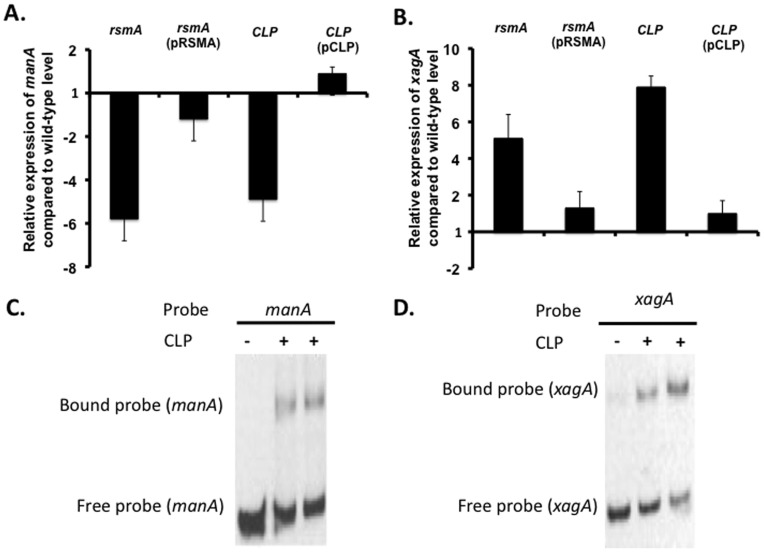
Clp divergently regulates the expression of *manA* and *xagA* but binds to the promoter region of both genes. (A, B) Expression of genes encoding *manA* and *xagA* was measured in the wild-type strain (8004), *rsmA* and *clp* mutant backgrounds by qRT-PCR as described in the *Methods*. Bacterial strains were grown to an OD at 600 nm of 0.8 in complex medium before RNA extraction. All qRT-PCR results were normalized using the *Ct*s obtained for the 16S rRNA amplifications run in the same plate. The relative levels of gene transcripts were determined from standard curves. The results shown are the means and standard deviations of triplicate measurements (C, D) Binding of Clp protein to the *manA* and *xagA* promoter region was assessed using EMSA. Each lane contained 1.5 nM DIG-labelled Probe DNA with in addition 20 µM purified GST (lane 1); 20 µM purified GST-tagged Clp (lane 2); 25 µM purified GST-tagged Clp (lane 3).

These previous observations were extended to examine expression of both *manA* and *xagA*, which is required for biofilm formation, in both *rsmA* and *clp* mutants using qRT-PCR. As expected, the level of *manA* transcript in both *rsmA* and *clp* mutants was reduced compared to the wild-type (Fig. 7A). Complementation restored *manA* transcript levels towards wild-type (Fig. 7A). In contrast, mutation of either *rsmA* or *clp* led to increased transcription of *xagA* (Fig. 7B). Complementation restored *xagA* transcript levels towards wild-type (Fig. 7B).

EMSA analysis showed that Clp bound to the promoter of both the *xagA* and *manA* genes in the absence of cyclic di-GMP (Fig. 7C,D). As expected, addition of the dinucleotide inhibited binding to both promoters (Fig. S3 and data not shown). These observations suggested that Clp has divergent action at different promoters, acting as an activator of *manA* but a repressor of *xag*.

**Figure 8:**
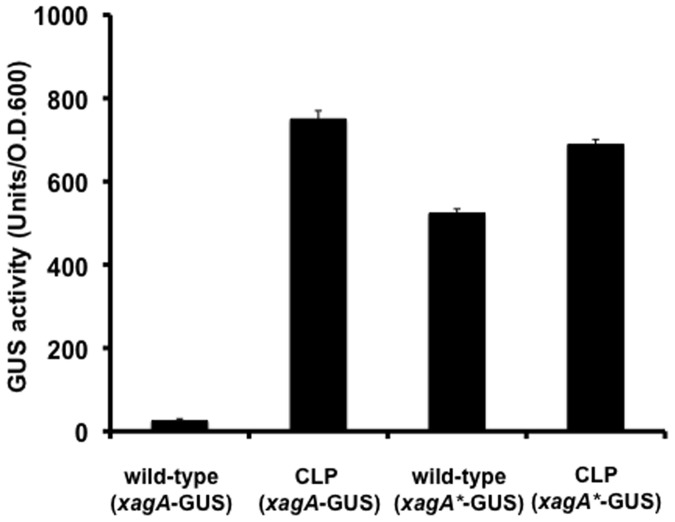
The transcription of *xagA* is negatively regulated by Clp. The action of Clp as a repressor of *xagA* was investigated using a *xag-gusA* transcriptional fusion. β-glucuronidase (GUS)-activity was determined in the wild-type and *clp* mutant as described in the *Methods*. The GUS activity was higher in the *clp* background than in wild-type background, consistent with role for Clp as a repressor. A *xagA-gusA* transcriptional fusion in which the putative binding site for Clp was altered by site directed mutagenesis to AAAAA-N6-TCGAT (to generate the xagA*-gusA transcriptional fusion) gave higher GUS-activity in the wild-type than seen with the native reporter, indicating a role for Clp binding in repression. In the *clp* mutant background, GUS activity directed by *xagA-gusA* and xagA*-gusA were similar. Bars show mean values and standard deviation from three independent experiments.

The possible action of Clp as a repressor of *xag* gene expression was further investigated using a *xag-gusA* fusion in which the promoter of *xagA* (211 nt) drives expression of *gusA* (see *Methods*). This construct was introduced into the wild-type and *clp* mutant. The levels of beta-glucuronidase were higher in the *clp* mutant compared to the wild-type background (Fig. 8). Complementation of the *clp* mutant led to restoration of *gusA* expression to levels seen in the wild-type background (Fig. S4). This experiment was repeated with a *xagA* promoter in which the putative binding site for Clp (TGTGA-N6-TCGAT) was altered by site directed mutagenesis to AAAAA-N6-TCGAT as described in *Methods*. This alteration prevented Clp binding to the promoter as measured by EMSA and led to higher levels of beta-galactosidase in the wild-type background than those seen with the native promoter (Fig. 8). In the *clp* mutant background however, both native and altered promoters gave similar levels of beta-galactosidase. Taken together, the findings are consistent with an action of Clp as a repressor of *xag* gene expression, where cyclic di-GMP binding relieves repression.

## Discussion

**Figure 9:**
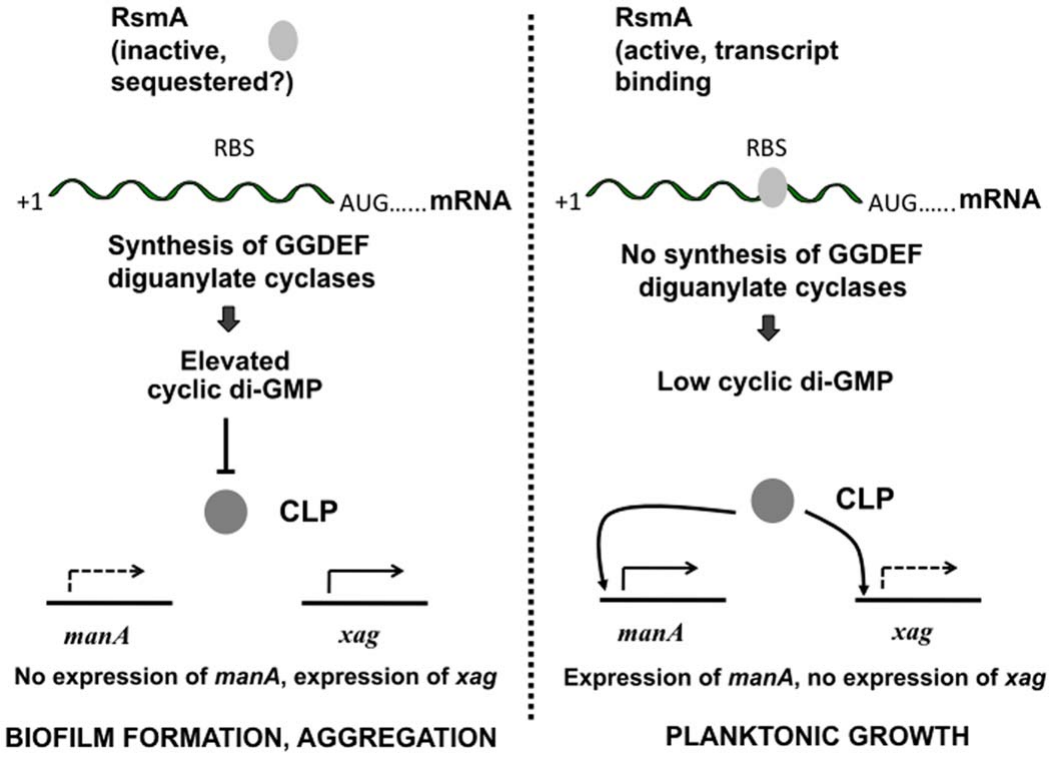
Model of regulation of biofilm formation and aggregation in *Xcc* by RsmA. The post-transcriptional regulator RsmA influences the expression of three genes encoding GGDEF domain proteins (*XC_1803, XC_2715* and *XC_2866*) by binding to the transcripts. RsmA binding inhibits translation of these mRNAs. Inactivation of RsmA, perhaps by sequestration by small RNA species, allows translation to occur, leading to elevated cellular levels of cyclic di-GMP. The binding of cyclic di-GMP to the transcription factor Clp prevents DNA binding. This reduces expression of *manA*, which requires Clp for expression but activates expression of the *xag* genes for which Clp is a repressor. Since ManA promotes biofilm dispersal and the *xag* gene products promote biofilm formation, these effects contribute to enhanced biofilm formation and aggregation. Conversely, when RsmA binds to the transcripts of *XC_1803, XC_2715* and *XC_2866,* the cellular levels of cyclic di-GMP are lower, *manA* expression is favoured and *xag* gene expression is reduced. This leads to biofilm dispersal and planktonic growth. We do not exclude the possibility that RsmA has additional direct or indirect effects on *manA* or *xag* gene expression or that regulation by Clp is more indirect, for example by positive regulation of a distinct repressor protein or that other functions regulated by cyclic di-GMP also have a role.

The work in this manuscript provides insight into the mechanisms by which the post-transcriptional regulator RsmA exerts a regulatory influence on biofilm formation in *Xcc*. The findings support a model by which RsmA influences the expression of three genes encoding GGDEF domain proteins that leads to alteration in cellular levels of cyclic di-GMP and consequent divergent effects on the expression of *manA* and *xag* mediated by the Clp transcriptional regulator (Fig. 9). The influence of RsmA on expression of specific GGDEF domain proteins is similar to that described previously in *E. coli* and *Salmonella*, where the RsmA homolog CsrA has been shown to directly influence expression of GGDEF domain proteins (Jonas et al., 2008; 2010). We do not exclude the possibility that RsmA has additional direct or indirect effects on *manA* or *xagA* gene expression however, although RsmA does not affect transcription of the *clp* gene (Fig. S4).

An intriguing observation in the current work is that mutation of *clp* leads to a down-regulation of expression of *manA* [5 and data not shown], that is associated with biofilm dispersal but an upregulation of *xag* gene expression, which is involved in biofilm formation [6]. The most parsimonious explanation for this finding is that Clp can act both as an activator and repressor of transcription of different genes. Similarly, other members of the CRP-FNR family (to which Clp belongs) have been shown to act both as activators and repressors [27], [28]. The effects of mutation of the putative Clp box on expression of the *xag*-*lacZ* reporter is consistent with a direct role for Clp as a repressor but we cannot exclude the possibility that regulation of *xag* gene expression by Clp is more indirect, for example by positive regulation of a distinct repressor protein. It is noteworthy that mutation of *clp* does not lead to an aggregated phenotype in *Xcc* ([29]; and data not shown). This suggests that regulation of Clp is necessary, but not sufficient for biofilm formation and that other cyclic di-GMP-influenced processes are also required.

The formation of biofilms in many bacteria is responsive to diverse environmental cues or stimuli and several elements in the proposed regulatory pathway are likely to be responsive to such cues. A body of work on different bacteria has shown that small RNAs modulate the activity of RsmA (CsrA) by sequestration to prevent binding to transcripts of target genes. The small RNAs that are presumed to fulfil this role in *Xcc* have yet to be described. Importantly, in other bacteria, the synthesis of these regulatory RNAs is regulated by two component systems that recognise as yet unknown signals. RsmA in *Xcc* regulates expression of three GGDEF domain proteins, two of which contain additional domains: XC_1803 contains a GAF domain and XC_2715 has a putative periplasmic binding domain. This suggests that the enzymatic activities of these proteins in cyclic di-GMP synthesis are responsive to environmental cues.

The Rpf/DSF cell-cell signalling system also influences biofilm dispersal and inversely regulates *manA* and *xag* gene expression. The Rpf/DSF system is linked to alterations in the level of cyclic di-GMP through the action of RpfG as a cyclic di-GMP phosphodiesterase. However RsmA does not influence DSF signalling in *Xcc* and Rpf/DSF does not act to regulate expression of *XC_1803*, *XC_2715* or *XC_2866*. This suggests that the DSF-dependent and RsmA-dependent pathways controlling biofilm dynamics intersect at the level of cyclic di-GMP, although other levels of interplay are possible.

In conclusion, the work described in this paper adds to an understanding of mechanisms by which *Xcc* controls biofilm formation in response to diverse environmental signals [5], [6]. Our knowledge of these processes remains fragmentary however and there a large number of questions that remain to be answered. For example, what is the substrate for ManA and what is the polymer whose synthesis is directed by the Xag proteins? What other factors are required for biofilm formation? Finally (and of particular relevance to the work described here), what are the small RNAs that we presume to act to modulate RsmA action and how are they in turn regulated?

## Methods

### Bacterial Strains, Plasmids and Growth Conditions

Bacterial strains and mutants used in this study are listed in Table S1. Bacterial culture conditions have been previously described (Barber et al., 1997; Slater et al., 2000). L medium contains Bactotryptone (Difco), 10 g/l; yeast extract, 5 g/l; sodium chloride, 5 g/l; and D-glucose, 1 g/l. NYGB medium contains Bacteriological Peptone (Oxoid, Basingstoke, U.K.), 5 g/l; yeast extract (Difco), 3 g/l; and glycerol, 20 g/l.

### General Molecular Biology Methods

Common molecular biological methods such as isolation of plasmid and chromosomal DNA, polymerase chain reaction (PCR), plasmid transformation as well as restriction digestion were carried out using standard protocols (Sambrook *et al.*, 1989). PCR products were cleaned using the QIAquick PCR purification kit (Qiagen) and DNA fragments were recovered from agarose gels using QIAquick mini-elute gel purification kit (Qiagen). Primer sequences are provided in Table S2.

### Cellular Quantification of Cyclic di-GMP

Cyclic di-GMP was quantified as described previously [11] in bacterial strains grown to an OD at 600 nm of 0.8 in NYGB medium.

### Construction of *gusA* Reporter Plasmids

Reporter plasmids pG1803, pG1824, pG2228, pG2715 and pG2866 were constructed by cloning the putative promoter region and ribosome binding site (∼500-bp region upstream of the start codon) of *XC_1803*, *XC_1824*, *XC_2228*, *XC_2715* and *XC_2866* respectively into the broad-host-range cloning vector pL6gus (Table S1), which harbors the coding region (without promoter and RBS) of β-glucuronidase (*gusA*) gene in its MCS (multiple cloning site). The putative promoter region and RBS of *XC_1803*, *XC_1824*, *XC_2228*, *XC_2715* and *XC_2866* were amplified from the chromosomal DNA of *Xcc*8004 using the primer pairs detailed in Tabel S2.

The *xagA* reporter plasmid pGUSXAGA was constructed by cloning the intergenic region 211 bp before the *xagA* gene into the broad-host-range cloning vector pLAFRJ, which harbours the promoterless β-glucuronidase (*gusA*) gene in its MCS (multiple cloning site). The 211 bp region upstream of the start codon (not including GTG) was amplified by PCR using the total DNA of the wild-type strain 8004 as the template (primers available upon request). The amplified DNA fragment, confirmed by sequencing, was inserted 9 bp upstream of the promoterless *gusA* ATG start codon in the vector pLAFRJ to create the recombinant plasmid pGUSXAGA (Table S1). The recombinant plasmid obtained was further confirmed by restriction analysis and PCR. All reporter plasmids were introduced into the *Xcc* strains of interest through conjugation as previously described [30].

### RNA Isolation and Quantitative Reverse Transcriptase Polymerase Chain Reaction (qRT-PCR) for Transcription Analysis

RNA was isolated from *Xanthomonas* strains of interest as previously described (McCarthy et al., 2008). Expression of the genes *XC_1803*, *XC_1824*, *XC_2228*, *XC_2715* and *XC_2866* were monitored in both wild-type and *rsmA* mutant backgrounds. Bacteria were grown to an OD_600_ nm of 0.8 in NYGB media and RNA was sampled. Total RNA (5 µl per sample) was reverse transcribed to produce single-stranded cDNA using Avian Myeloblastosis Virus (AMV) reverse transcriptase (RT) (Promega), and primers for the genes respectively (see Table S2). For RT-PCR, the total RNA was heated at 70°C for 10 min and reverse transcribed in a reaction mixture (0.5 mM deoxynucleoside triphosphate mixture (dNTP), 10 U/µl of SuperScript II reverse transcriptase (Invitrogen-Life Technologies), 10 ng/µl random primers, and 3 mM MgCl_2_) at 42°C for 50 min. Amplification was then performed with oligonucleotides detailed in Table S2. As a control, RT-PCR was similarly applied to analyse expression of the 16S rRNA gene. Total RNA (5 µl per sample) was reverse transcribed to give cDNA, using primers detailed in Table S2. Quantitative RT-PCR protocols were used as described previously [30], [31].

### Over-expression and Purification of GST-tagged Clp

For purification of proteins that were fused to the glutathione *S*-transferase (GST), gel filtration was carried using a Sephadex G-75 (Sigma) as previously described [9], [10]. Protein concentration was assayed using NanoDrop®. Purified proteins were stored at –20°C.

### Electrophoretic Gel Mobility Shift Assays

The DNA probes used for EMSA were prepared by PCR amplification of the desired *manA* and *xagA* upstream regions, using oligonucleotides as the primers (primers available upon request). The Clp protein, the binding conditions, and detection procedures were as previously described [32]. Briefly, the purified PCR products were 3′-end-labelled with digoxigenin following the manufacturer’s instruction (Roche). The EMSA was carried out using the DIG Gel Shift Kit 2nd Generation (Roche) as recommended by the manufacturer with some modifications. Ten fmoles of the DIG-labelled fragment and a range of 0–50 µM of Clp protein were added to the binding reaction. The mixture was allowed to proceed at room temperature for 45 min. The samples were separated by electrophoresis on 6% native polyacrylamide gels and transferred to Hybond-N blotting membrane (Amersham). Protein–DNA complexes were visualized by NBT/BCIP according to the manufacturer’s instructions (Roche).

### RNA Gel Mobility Shift Assays

RNA gel mobility shift assays were carried out as previously reported by Yakhnin et al., [33]. Briefly, DNA templates for the generation of the targeted RNA transcripts were produced using wild-type strain 8004 DNA template and the specific primer pairs listed in Table S2. These DNA fragments were transcribed into RNA using the MEGAshortscript kit (Ambion) following the manufacturer’s instructions. After gel purification, the 3′ ends of these transcripts were labeled with biotin using the Pierce RNA 3′ End Biotinylation Kit (Thermo Fisher Scientific). Biotin-labeled RNA was gel-purified and re-suspended in TE (10 mM Tris-HCl pH 8.0, 1 mM EDTA), heated to 85°C and chilled on ice. Then 400 nM His_6_-RsmA protein and 80 pM Biotin-labeled RNA were added into a tube containing 10 µl of binding reaction systems [10 mM Tris-HCl pH 7.5, 10 mM MgCl2, 100 mM KCl, 3.25 ng total yeast RNA, 20 mM DTT, 7.5% glycerol, 4 U SUPERasin (Ambion, Austin, TX)], and mixed well. The mixtures were incubated at 28°C for 30 min to allow RsmA–RNA complex formation. Binding reaction mixtures were separated using 12% native polyacrylamide gels, and signal bands were visualized with using the Detector™ AP Chemiluminescent Blotting Kit (KPL, Inc., Gaithersburg, MD, USA) according to the manufacturer’s instructions.

### Biofilm Assays

Biofilm was assessed by: (i) Aggregation in L medium as described previously [3] and (ii) Attachment to glass was determined by crystal violet staining. Where log-phase-grown bacteria were diluted to OD600 nm = 0.02 in L media broth, and 5 ml was incubated at 30°C for 24 h in 14-ml glass tubes. After gently pouring off the media, bacterial pellicles were washed twice with water and were then stained with 0.1% crystal violet. Tubes were washed and rinsed with water until all unbound dye was removed [21], [24], [34]. Three independent assays were carried out for each strain.

## Supporting Information

Figure S1 **Domain organization of the various GGDEF, EAL, GGDEF-EAL domain-containing proteins, identified by analysis of the annotated genome sequence of** ***Xcc*** **8004.** Domains were assigned according to Pfam and SMART (http://smart.embl-heidelberg.de/).
(TIF)

Figure S2 **The isolated GGDEF domains of XC_1803, XC_2715 and XC_2866 possess cyclic di-GMP cyclase activity.** The DNA encoding the GGDEF domain from XC_1803, XC_2228 or XC_2866 with a C-terminal His6 tag was cloned by PCR using oligonucleotide primers described in Table S2. The His-6-tagged proteins were purified by nickel affinity chromatography and analyzed by SDS/polyacrylamide gel electrophoresis with Coomassie blue staining using similar methods as described previously (Ryan *et al*., 2006; 2010). Lane 1, molecular mass markers; lane 2, XC_1803 (∼20 kDa); lane 3, XC_2715 (∼20 kDa) and XC_2866 (∼35 kDa). (*B*) Di-guanylate cyclase activity of the GGDEF domains from XC_1803, XC_2228 and XC_2866. GTP (top; 0 min) and products (bottom; 60 min) of the reaction, separated by reversed-phase HPLC. The standard reaction mixture (total volume, 200 µl) contained 5 µM enzyme in 50 mM Tris-HCl (pH 7.6), 10 mM MgCl2, 0.5 mM EDTA, and 50 mM NaCl. The reaction was started by the addition of 50 µl of substrate (final concentration, 150 µM) to the prewarmed reaction mixture and was carried out for 60 min. The mixture was immediately placed in a boiling water bath for 3 min, followed by centrifugation at 15,000×*g* for 2 min. The supernatant was filtered through a 0.22 µm-pore-size filter and analyzed by high-pressure liquid chromatography (HPLC), as described previously [9]. The presence or absence of cyclic di-GMP in the HPLC fractions was confirmed by mass spectrometry. Y-axis although not shown are units of milliabsorbance.
(TIF)

Figure S3 **EMSA assessment of the impact of cyclic di-GMP on Clp binding to the** ***xagA*** **promoter.** DIG-labelled promoter fragments were incubated with purified Clp protein in the absence or presence of nucleotide, as indicated.
(TIF)

Figure S4 **The expression of the** ***clp*** **gene is unaltered in an** ***rsmA*** **mutant.** Transcript level of the gene that encodes the Clp protein was measured in wild-type strain (8004) and *rsmA* mutant backgrounds by qRT-PCR as described in the *Methods*. Bars show mean fold changes obtained from three independent experiments.
(TIF)

Table S1 Bacterial strains and plasmids used in this study.
(DOCX)

Table S2 Primers used throughout this study.
(DOC)
