# Supplementary figures and images for: RsmA Regulates Biofilm Formation in Xanthomonas campestris through a Regulatory Network Involving Cyclic di-GMP and the Clp Transcription Factor
Source: PLoS One. 2012 Dec 21;7(12):e52646. doi: 10.1371/journal.pone.0052646 (PMC3528676; doi:10.1371/journal.pone.0052646)

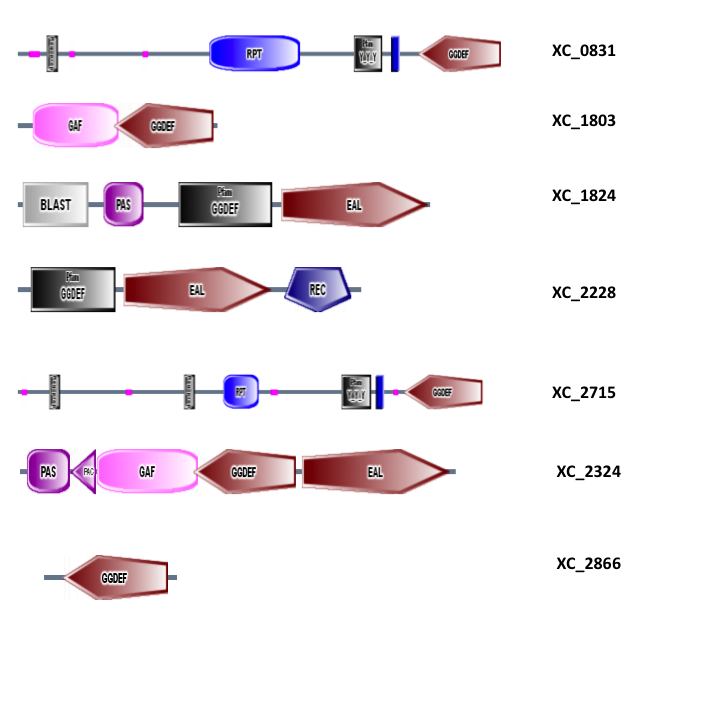

Supplement: Figure S1 — Domain organization of the various GGDEF, EAL, GGDEF-EAL domain-containing proteins, identified by analysis of the annotated genome sequence of Xcc 8004. Domains were assigned according to Pfam and SMART (http://smart.embl-heidelberg.de/). (TIF) [file pone.0052646.s001.tif]
