# Supplementary material for: RsmA Regulates Biofilm Formation in Xanthomonas campestris through a Regulatory Network Involving Cyclic di-GMP and the Clp Transcription Factor
Source: PLoS One. 2012 Dec 21;7(12):e52646. doi: 10.1371/journal.pone.0052646 (PMC3528676; doi:10.1371/journal.pone.0052646)
Supplement: Table S1 — Bacterial strains and plasmids used in this study. (DOCX) [file pone.0052646.s005.docx]

**Supporting Table S1.** Bacterial strains and plasmids used in this study.

| **Strains or plasmids** | **Relevant characteristics** | **Reference or source** |
| --- | --- | --- |
| ***Escherichia coli*** |  |  |
| JM109 | *RecA*1, *endA*1, *gyrA*96, *thi*, *supE*44, *relA*1  △ (*lac*-*proAB*)/F’ [*traD*36, *lacI*^q^, *lacZ* △M15] | Yanisch-Perron et al., (1985) |
| BL21(DE3)pLysSBlrsmA | F^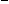^ *ompT hsdS_B_* (r_B_^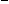^m_B_^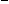^) *gal dcm* (DE3) pLysS  BL21(DE3)pLysS harboring pETrsmA, Kan^r^ | Novagen, Germany |
|  |  |  |
| ***Xanthomonas campestris*** |  |  |
| 8004 | wild-type, Rifr | Daniels *et al*., (1984) |
| *rsmA* | *rsmA* (XC_2506) has been deleted, Rifr | Chao *et al*., (2008) |
| *rsmA* (pRSMA) | *rsmA* mutant harboring pLRSMA_,_ Rifr, Tcr | Chao *et al*., (2008) |
| *XC_1803* | Mutant created with pK18*mobkan* XC_1803::Kmr, Rifr | Ryan et al., (2007) |
| *XC_2228* | Mutant created with pK18*mobkan* XC_2228::Kmr, Rifr | Ryan et al., (2007) |
| *XC_2866* | Mutant created with pK18*mobkan* XC_2866::Kmr, Rifr | Ryan et al., (2007) |
| *XC_1803/XC_2228* | Mutant created with pK18*mobkan* and Tn5*gus*A5 XC_1803::Kmr,Tcr, Rifr | Ryan et al., (2007) |
| *XC_2228/XC_2866* | Mutant created with pK18*mobkan* and Tn5*gus*A5 Kmr,Tcr, Rifr | Ryan et al., (2007) |
| *XC_1803/XC_2228/XC_2866* | Mutant created with pK18*mobkan* and Tn5*gus*A5 Kmr,Tcr, Rifr | Ryan et al., (2007) |
| *CLP* | *CLP* (XC_0486) has been deleted. | Chin et al., (2010) |
| CLP (pCLP) | *CLP* mutant harboring pLCLP_,_ Rifr, Tcr | Chin et al., (2010) |
| **Plasmids and expression constructs** |  |  |
| pET-30a-C(+) | Expression vector, allow the production of fusion proteins containing amino terminal 6xHis-taggted sequences. Kanr | Novagen, Germany |
| pETrsmA | pET-30a-C(+)containing the coding region of the *Xcc* *rsmA* gene. Kanr | This study |
| pL6gus | pLAFR6 containing a promoter-less-*gusA* gene; Tcr | Feng et al., (2009) |
| pRSMA | pLAFR3 containing the coding region of the *Xcc* *rsmA* gene. Kanr | Chao *et al*., (2008) |
| pLAFRJ | Broad-host-range cloning vector; Tcr | Feng et al., (2009) |
| pLCLP | pLAFR3 containing the coding region of the *Xcc* *CLP* gene. Kanr | This study |
| pG1803 | pL6gus expressing the promoter region of *XC_1803* | This study |
| pG1824 | pL6gus expressing the promoter region of *XC_1824* | This study |
| pG2228 | pL6gus expressing the promoter region of *XC_2228* | This study |
| pG2715 | pL6gus expressing the promoter region of *XC_2715* | This study |
| pG2866 | pL6gus expressing the promoter region of *XC_2866* | This study |
| pGUSXAGA | pLAFRJ expressing the *xagA promoter region fused to gusA* | This study |
| pGUSXAGA* | pLAFRJ expressing the *xagA* promoter region fused to gusA* (promoter region has an alteration in the putative CLP binding site) | This study |

Key to antibiotic resistances: Rif: rifampicin, 50 µg/ml; Sp: spectinomycin 50 µg/ml; Tc: tetracycline 20 µg/ml; Km: kanamycin, 15 µg/ml.

**Reference**

Chao NX., Wei K., Chen Q., Meng QL., Tang DJ., He YQ., Lu GT., Jiang BL., Liang XX., Feng JX., Chen B., Tang JL. 2008. The *rsmA*-like gene *rsmA_Xcc_* of *Xanthomonas campestris* pv. *campestris* is involved in the control of various cellular processes, including pathogenesis. Mol Plant Microbe Interact. **21:** 411-423.

Chin KH., Lee YC., Tu ZL., Chen CH., Tseng YH., Yang JM., Ryan RP., McCarthy Y, Dow JM, Wang AH, Chou SH.(2010) The cAMP receptor-like protein CLP is a novel c-di-GMP receptor linking cell-cell signaling to virulence gene expression in *Xanthomonas* *campestris*. *J Mol Biol.* **396:** 646-662.

Daniels, M. J., Barber, C. E., Turner, P. C., Cleary, W. G., and Sawczyc, M. K. 1984. Isolation of mutants of *Xanthomonas campestris* pathovar *campestris* showing altered pathogenicity. J. Gen. Microbiol. **130:** 2447-2455.

Ryan RP., Fouhy Y., Lucey JF., Jiang BL., He YQ., Feng JX., Tang JL., Dow JM. (2007) [Cyclic di-GMP signalling in the virulence and environmental adaptation of *Xanthomonas campestris.*](http://www.ncbi.nlm.nih.gov/pubmed/17241199) *Mol Microbiol*. **63:** 429-42.

Yanisch-Perron, C., Vieira, J., and Messing, J. 1985. Improved M13 phage cloning vectors and host strains: nucleotide sequences of the M13mp18 and pUC19 vectors. Gene **33:** 103-119.
