# Supplementary material for: RsmA Regulates Biofilm Formation in Xanthomonas campestris through a Regulatory Network Involving Cyclic di-GMP and the Clp Transcription Factor
Source: PLoS One. 2012 Dec 21;7(12):e52646. doi: 10.1371/journal.pone.0052646 (PMC3528676; doi:10.1371/journal.pone.0052646)
Supplement: Table S2 — Primers used throughout this study. (DOC) [file pone.0052646.s006.doc]

**Supporting Table S2. Primers used throughout this study.**

| **Primer pair** | **sequence (5’ to 3’)^a,b^** | **Product length (bp)** |
| --- | --- | --- |
| **For EMSA** |  |  |
| 0249EMF/0249EMR | **taatacgactcactataggg**tctctttgtctgtgagaaca/ttttggcaacgcttccagtc | 175 |
| 0362EMF/0362EMR | **taatacgactcactataggg**cgcccggcccaaagaatcct/atacatgccggggcggagtt | 163 |
| 0420EMF/0420EMR | **taatacgactcactataggg**ttactgcggtcgcgtctgca/tggggtgttcgggctgttct | 145 |
| 0613EMF/0613EMR | **taatacgactcactataggg**agcgcacggcgtggtggttt/tcgagcagcatggcttcggt | 129 |
| 0637EMF/0637EMR | **taatacgactcactataggg**ggctcaagttgcccgtcgccttgcc/aaggcgtcgttggcggggat | 140 |
| 0641EMF/0641EMR | **taatacgactcactataggg**agcctgcctgtcaccggtgt/agatcgagcagatcggcgtc | 146 |
| 0675EMF/0675EMR | **taatacgactcactataggg**gtgaatcgacgcatttgcgc/gcatccatgtctccggcaaa | 132 |
| 0831EMF/0831EMR | **taatacgactcactataggg**tccacctagcgcgtcgctgc/agctcgcctgtgtgcgcatg | 152 |
| 1036EMF/1036EMR | **taatacgactcactataggg**attgtttcaatgagttaaaa/atcggggcattgctgctgat | 164 |
| 1383EMF/1383EMR | **taatacgactcactataggg**atccggattgcacgcgcggc/ttgccgccactgcttgcttg | 150 |
| 1411EMF/1411EMR | **taatacgactcactataggg**tcctgctggatgccgacgcc/gccagtgcgcagttgcagga | 158 |
| 1476EMF/1476EMR | **taatacgactcactataggg**actgggcgcctgagacgcat/atcaacagcgccaacggcag | 151 |
| 1582EMF/1582EMR | **taatacgactcactataggg**ggtcgcgccgccggatgatc/gcgatgcgcgtatgcagtcg | 130 |
| 1755EMF/1755EMR | **taatacgactcactataggg**ggacgacttcgatttccgcc/atcgtcaacgcagagcagggga | 153 |
| 1766EMF/1766EMR | **taatacgactcactataggg**accgagccgttagctgacct/tggtgaacgtgcgcgagttc | 154 |
| 1803EMF/1803EMR | **taatacgactcactataggg**cggtaccacgctcgatgcaga/ttgtaactgtgcagcgcggc | 165 |
| 1824EMF/1824EMR | **taatacgactcactataggg**ggaagcgtggcctaggtctc/ttgcctttctgcatgcgcgg | 134 |
| 1841EMF/1824EMR | **taatacgactcactataggg**gctttttcggcaccacggcg/agcgcacctgcgtcattcca | 136 |
| 2161EMF/2161EMR | **taatacgactcactataggg**aaatgcctcatcgtgtctcg/atgccattgagctccgtggt | 148 |
| 2226EMF/2226EMR | **taatacgactcactataggg**cgaaagcggcggcatggcgg/ttcccagcggctggtgccat | 177 |
| 2228EMF/2228EMR | **taatacgactcactataggg**cgcgatagaatcggctcc/ttgacgatttccagtccgggcg | 161 |
| 2274EMF/2274EMR | **taatacgactcactataggg**accggcctcaagctttaggtgc/cagcgtgggcatcgtttcct | 132 |
| 2275EMF/2275EMR | **taatacgactcactataggg**aagaaagccgtcactgcagccg/tcaaggccggtgcatcatgc | 119 |
| 2276EMF/2276EMR | **taatacgactcactataggg**ctcggttgcatgaaccccgt/cgacagcagcaccagcgaaa | 159 |
| 2324EMF/2324EMR | **taatacgactcactataggg**accgcacctcccccgcatccagcc/gaacgggtgtccgacgcgctct | 151 |
| 2335EMF/2335EMR | **taatacgactcactataggg**catatgctagggcggagctt/ccgccggattccctaaaaca | 148 |
| 2459EMF/2459EMR | **taatacgactcactataggg**gattgccgcgctgcaccact/gcgtggaatgcatgtgccca | 125 |
| 2715EMF/2715EMR | **taatacgactcactataggg**cggcaagcccactacaccag/ttgcgccatccccactatcg | 169 |
| 2793EMF/2793EMR | **taatacgactcactataggg**ccaccgggcaagcgaagtga/cggcgtggcgttttgctttt | 129 |
| 2795EMF/2795EMR | **taatacgactcactataggg**aacgttgcgcacgcacgatg/aatggaggatcacgggcgaa | 133 |
| 2866EMF/2866EMR | **taatacgactcactataggg**atgccgagcgctttgaaaag/tcgcgtgcctgttcgaagat | 143 |
| 2946EMF/2946EMR | **taatacgactcactataggg**tcgcaactcctgcatgcacc/cgccgcgaggtaaaggaaga | 145 |
| 3163EMF/3163EMR | **taatacgactcactataggg**ggtggtgatggcctgcaaca/tgggaaagtcctggccatcg | 162 |
| 3800EMF/3800EMR | **taatacgactcactataggg**cgcctcttcggattcagatt/cgccgcaaaatagggaacag | 156 |
| 3829EMF/3829EMR | **taatacgactcactataggg**tgatcgaacgcacgccgatg/tccaccgcgatgcattcacc | 135 |
| 3962EMF/3962EMR | **taatacgactcactataggg**catacgccacagcgcagacc/tgcggtgccgcttcacttca | 136 |
| 4313EMF/4313EMR | **taatacgactcactataggg**agcggcacatctgcgtggca/cgtggcgctgtttcaacgtg | 131 |
| **For protein purification** |  |  |
| 2506PF/2506PR | cgcggatccatgttgatcctcactcgccga/agcaagctttcagtccgaacaatcgtcgtt | 232 |

| **For RT-PCR** |  |  |
| --- | --- | --- |
| 0249RTF/0249RTR | catgccttgatgaccgccgaagaact/catcaggtcttcgccgaaatccaggtc | 162 |
| 0362RTF/0362RTR | atcttcgaagacggccgtagcgtggt/cgccatcgaatgcaggtaggtgtagtcg | 192 |
| 0420RTF/0420RTR | cgctactgatccgcctgttttcgcat/tgtatttgtcgatcagctggcgcagg | 183 |
| 0613RTF/0613RTR | ctcgagctggatgcgctgacacaact/cgaggcaccgtattcgtattcgatcagg | 168 |
| 0637RTF/0637RTR | gtgtcgttctgctcgtacacggtgatgg/gagcatgcacaaggtgccgtaggcata | 177 |
| 0641RTF/0641RTR | cgaagtgtgtgcggccgaagaattgt/ttccatcatcgggtagaccagcagcg | 220 |
| 0675RTF/0675RTR | gtcggcatccatctgcctttgttgttg/tgggcaagcgttgatcggcgtagtac | 214 |
| 0831RTF/0831RTR | gcatccgctcaagcacaccgcctatca/cacggtgaaatcgcgcccattgaagc | 163 |
| 1036RTF/1036RTR | ggttcagcatgaggacgaactacgcacc/cgaacgaactccgcattgcagtactgaat | 138 |
| 1383RTF/1383RTR | accatgaccatcgccgatgcggtgtt/actgaccagaaacgcatgcatcgccg | 105 |
| 1411RTF/1411RTR | cgaaggtttgatccacgcactcaccc/acaggttcaaggccagcgtgagttgc | 107 |
| 1476RTF/1476RTR | cagtccttgcgtgccgaactgcatct/ggtcaccatcacatcgtgcagcatgc | 114 |
| 1582RTF/1582RTR | gctgtcgcctgaaggcgattttctgacc/tgtgttgatccaaggccaccacaccg | 137 |
| 1755RTF/1755RTR | cagcatcctgcacaagcccgataccct/attcgtggtggtgcagggcgatatcg | 146 |
| 1766RTF/1766RTR | gtgctggacgaagaaagtggctggttcc/ttcgtcgtagacgccactgaccaccac | 135 |
| 1803RTF/1803RTR | gatggcgttgatctcgctgatcgacca/acttccatcatctggtcgggctgcagg | 135 |
| 1824RTF/1824RTR | cgaaggccagtccggcttcctgttgat/acaacgcaaagctgtgctcgccgaag | 173 |
| 1841RTF/1841RTR | gcaagatcgaatccagcgccaacctc/tagatggccagcaggattcctagcgcc | 127 |
| 2161RTF/2161RTR | gcacgtttggtatgccattcgctggg/ttgtcttcaaaggccgccacggtctg | 137 |
| 2226RTF/2226RTR | accgacgtgcatcatcataaattgctcg/cacccacaattggtccttcaaggtgtcc | 129 |
| 2228RTF/2228RTR | tggaaatcgtcaagccgacccgtctg/atatggaagatcatcagcggcgggcc | 103 |
| 2274RTF/2274RTR | cagccaggacctgttccagctcgactt/atgacctttgagaccagcgggtcggt | 147 |
| 2275RTF/2275RTR | tgctgttggtactgctgtggctggcgt/cagcaatgtgttgcgggtctgcagca | 137 |
| 2276RTF/2276RTR | catctggtcgatgcatttcgtcggca/aggtgatctccggatccatgcgcatc | 248 |
| 2324RTF/2324RTR | gcaagagcgcccgctaatcgaagtga/gatcttcaggccattgaccgcatcgg | 172 |
| 2459RTF/2459RTR | tcctgcacggtctttgcctgctggact/ttcgtccaccaaggagaccagggcgat | 116 |
| 2715RTF/2715RTR | acctacgccgatggcgtgtttgtgctg/gtcgatcacgattgcgcgaatgtgcc | 105 |
| 2793RTF/2793RTR | tgcgagcacggtctgcattccattgt/ttccagatagcggcggttgaacaggc | 220 |
| 2795RTF/2795RTR | ttcaaggacgtcaacgatctgtacgggc/cgaagcggaacacctgatagggctcac | 112 |
| 2866RTF/2866RTR | atgccgacgaatcggaaaaccgcatg/cgcatcgttgacgcgcttgaagtcgt | 191 |
| 2946RTF/2946RTR | ggatcaactgcatcatctggtcgacacg/aaacgcggtctggcgaatgcttgtac | 115 |
| 3163RTF/3163RTR | caccgccattaccatggcctttcaaccc/aagttgatcgacagcagcgccggcaac | 219 |
| 3800RTF/3800RTR | tctcgcagatcaaaacggaaccatcatg/tgcatgcggcaggtacttcgaaaagat | 109 |
| 3829RTF/3829RTR | agcggaattccgctgttcgatgccca/agatagcgcgcggcatggttgatcagg | 185 |
| 3962RTF/3962RTR | gcttcccaccggcgtgtcgtattacct/tcaacgatgtggtggctgctcagcac | 225 |
| 4313RTF/4313RTR | ggtggtcgatagcgaagccgaaccgtt/tcactgcgtcatcgaggatggcatgc | 200 |

^a^ Primers were designed according to the genome sequence of *Xcc*8004

^b^Underlined and bolded letters are added T7 promoter sequences, and boxed letters are the added restriction sites.
